# Impacts of pH and Base Substitution during Deaerator Treatments of Herring Milt Hydrolysate on the Odorous Content and the Antioxidant Activity

**DOI:** 10.3390/foods11131829

**Published:** 2022-06-22

**Authors:** Sarah Todeschini, Véronique Perreault, Charles Goulet, Mélanie Bouchard, Pascal Dubé, Yvan Boutin, Laurent Bazinet

**Affiliations:** 1 Department of Food Sciences and Laboratoire de Transformation Alimentaire et Procédés ÉlectroMembranaires (LTAPEM, Laboratory of Food Processing and ElectroMembrane Processes), Université Laval, Québec, QC G1V 0A6, Canada; sarah.todeschini.1@ulaval.ca (S.T.); veronique.perreault.5@ulaval.ca (V.P.); 2 Institute of Nutrition and Functional Foods (INAF), Université Laval, Québec, QC G1V 0A6, Canada; yvan.boutin@tbt.qc.ca; 3 Department of Phytology, Université Laval, Québec, QC G1V 0A6, Canada; charles.goulet@fsaa.ulaval.ca; 4 Investissement Québec-Centre de Recherche Industrielle du Québec (CRIQ, Quebec Investment–Industrial Research Center of Quebec), Québec, QC G1P 4C7, Canada; melanie.bouchard@invest-quebec.com (M.B.); pascal.dube@invest-quebec.com (P.D.); 5 Centre Collégial de Transfert de Technologie en Biotechnologie (TransBIOTech, College Center for Technology Transfer in Biotechnology), Lévis, QC G6V 6Z9, Canada

**Keywords:** herring milt hydrolysate, deodorization, deaerator, off-flavors, trimethylamine, dimethylamine, trimethylamine oxide, sensory analysis, antioxidant activity

## Abstract

Despite the biological interest in herring milt hydrolysate (HMH), its valorization is limited by its unpleasant odor resulting from the presence of mainly amine and carbonyl compounds. Recently, a deaerator was demonstrated as an interesting avenue to reduce the odorous content of HMH. However, the removal rate of amine and carbonyl compounds was highly dependent on the operating conditions, and the impact of such a process on the biological potential of HMH was not considered. Therefore, this study aimed to optimize the deaerator process by assessing the impacts of the combination of deaerator treatments at neutral and basic pH, the increase in pH from 10 to 11, and the substitution of NaOH by KOH on the odorous content and the antioxidant activity of HMH. Results showed that the highest deodorization rate of HMH was obtained when a deaerator treatment at neutral pH was combined with another one at basic pH using KOH for alkalization. This condition resulted in a decrease in the dimethylamine and trimethylamine contents by 70%, while certain compounds such as 2,3-pentanedione, methional, (*E*,*E*)-2,4-heptadienal, or (*E*,*Z*)-2,6-nonadienal were almost completely removed. Removal mechanisms of the targeted compounds were totally identified, and the performance of the developed process was confirmed by sensory analysis. Lastly, it was shown that the antioxidant potential of HMH was not affected by the deodorization process. These results demonstrated the feasibility of deodorizing a complex matrix without affecting its biological potential.

## 1. Introduction

The fish processing industry is of significant importance in many countries all over the world [1]. Over the past 20 years, the major growth in fish consumption has been responsible for the huge increase in by-products leading to environmental and disposal problems [1,2]. Fish by-products include mainly skin, head, bones, viscera, and milt, and they can account for 70% of the fish fresh weight [1]. In order to promote the valorization of these underutilized resources, their modification by chemicals or enzymes has been highly considered [1]. The resulting hydrolysates have received great interest since they have demonstrated a broad range of bioactivities including antioxidant, anticancer, antimicrobial, anti-inflammatory, antihypertensive, and antidiabetic properties [1]. Herring milt hydrolysate (HMH) has been reported to have antioxidant, anti-inflammatory, and antidiabetic activities [2,3,4,5,6]. Therefore, HMH and other fish by-product hydrolysates definitely have a huge potential to be used as new ingredients in the food, nutraceutical, pharmaceutical, and cosmetic sectors. Nevertheless, in spite of their biological interest, fish by-product hydrolysates including HMH present the major drawback of being associated to off-flavors [7]. Since odor is a major criterion regarding the acceptance of a product by consumers, there is an urgent need to overcome this main issue [7,8].

Off-flavors in fish products and related products, such as hydrolysates, are generally ascribed to several volatile, hydrophobic, and low-molecular-weight (<400 Da) compounds [7]. Among them, trimethylamine (TMA) and dimethylamine (DMA) represent the most well-known agents responsible for unpleasant odors [9]. Both TMA and DMA are generated by the breakdown of trimethylamine oxide (TMAO), an odorless substance occurring in marine fish species playing a major role in the osmoregulation and protein stabilization [9]. Nevertheless, off-flavors in fish materials are not only due to nitrogen-containing compounds since other chemical groups such as aldehydes, ketones, alcohols, or sulfur-containing compounds have also been identified as major sources of unpleasant odors [7]. Concerning HMH, the presence of TMA, DMA, and TMAO was recently demonstrated, in addition to carbonyls, as the major contributors to its odor [8].

To overcome off-flavors in marine resources, several deodorization strategies have already been proposed including fermentation, use of antioxidants, and adsorption processes [7,10,11,12,13]. Even if these strategies have demonstrated their relevance at different degrees, they all seem to be associated with drawbacks, thus hampering their extensive use. Recently, Todeschini et al. (2020, 2021) [8,14] demonstrated the potential of a deaerator to be used as a new HMH deodorization method. Indeed, they showed that the application of a deaerator treatment at pH 10 led to an 80% decrease in the TMA content of HMH, while the application of a deaerator treatment at neutral pH was particularly efficient to reduce the content of most potent odor-active compounds, belonging mainly to the carbonyl group [14]. On this basis, it would be of interest to assess if a combination of treatments at both neutral and basic pHs could enhance the performance of this deodorization method by targeting both carbonyl and amine compounds. Furthermore, in the study of Todeschini et al. (2021) [14], the deaerator treatment at pH 10 did not result in similar decreases in DMA and TMA, probably due to the fact that DMA (pKa ≈ 10.70) could be involved in electrostatic interactions with other HMH components, thus hampering its optimal removal by deaerator. Therefore, conducting a deaerator treatment at higher pH, such as pH 11, could be an avenue to release DMA from these potential interactions and increase its removal rate. Nonetheless, performing treatments under basic conditions implied the use of sodium hydroxide (NaOH), positively correlated with hypertension concerns due to the generation of Na^+^ species, to alkalize HMH. This issue could be addressed by substituting NaOH with other alkaline salts such as potassium hydroxide (KOH), but the impacts of this substitution on the deodorization performance should be evaluated. Lastly, no information has been reported regarding the potential effects of deaerator treatments, as well as alkaline conditions, on the bioactive potential of HMH. As mentioned before, HMH has been reported to demonstrate, in particular, antioxidant properties [2,3]. Since the aim of a deodorization method is to remove off-flavors without affecting the properties of the matrix, the impacts of such treatments should be better considered, especially those regarding the HMH antioxidant potential.

In this context, the present work aimed to optimize the deaerator deodorization method by (1) assessing the impacts of a combination of deaerator treatments at neutral and basic pH on the removal rate of the odorous content of HMH, (2) studying the effects of the substitution of NaOH by KOH on the odorous content of HMH and on the deaerator performance, (3) evaluating the consequences of an increase in pH from 10 to 11 on the DMA and TMA removal rate by deaerator treatment under alkaline conditions, (4) characterizing the impacts of such conditions on the odor of HMH, and (5) assessing the impacts of the deodorization treatments on the antioxidant properties of HMH.

## 2. Materials and Methods

### 2.1. Materials

#### 2.1.1. Chemicals

Hydrochloric acid (HCl), potassium hydroxide (KOH), and 2,2-diphenyl-1-picrylhydrazyl (DPPH) were purchased from VWR (Montréal, QC, Canada). Sodium hydroxide (NaOH), methylene chloride, and nonyl acetate were obtained from Fisher (Montréal, QC, Canada). Helium was from Praxair (Mississauga, ON, Canada). DMA, TMA, TMAO, and Trolox standards, as well as 2,2′-azobis(2-methylpropionamidine) dihydrochloride (AAPH) and fluorescein, were supplied by Sigma-Aldrich (St-Louis, MO, USA). 

#### 2.1.2. Herring Milt Hydrolysate (HMH)

HMH dry powder was obtained from Ocean NutraSciences (Matane, QC, Canada), stored at −30 °C under vacuum, and protected from light before its use. Its chemical composition included 79.27% ± 0.17% total nitrogen, 48.28% ± 0.44% peptides, 18.48% ± 1.27% lipids, 27.30% ± 3.57% nucleic acids, and 11.55% ± 0.20% ashes [2].

### 2.2. Methods

#### 2.2.1. Protocol

**Figure 1:**
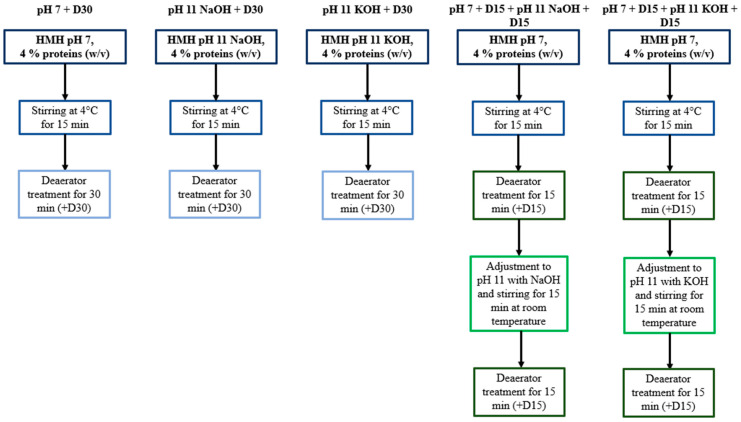
Summary of the experimental protocol.

HMH solutions, dissolved to 4% proteins (*w/v*), were adjusted either to pH 7 with 6 M HCl or to pH 11 with 6 M NaOH or 6 M KOH and were stirred for 15 min at 4 °C before being treated by an ERV2 deaerator (Koruma, Neuenburg, Germany). Treatments were performed in closed circuit under 100 Torr for 30 min according to Todeschini et al. (2020) [8]. For the conditions implying a first deaerator treatment at pH 7 followed by another one at pH 11, each deaerator treatment was conducted for 15 min to maintain a short processing time. Moreover, before undergoing the second 15 min deaerator treatment, the HMH solution was basified to pH 11 and then stirred for 15 min at room temperature. Samples were collected after the 15 min stirring at 4 °C and then at each step of the protocol. They were stored at −30 °C and protected from light, before being further analyzed in terms of volatile compound contents and antioxidant activity. All the conditions (Figure 1) were tested in triplicate and in a random order.

#### 2.2.2. Analyses

a. Volatile Compound Analyses

*DMA, TMA, and TMAO*

DMA, TMA, and TMAO contents were determined according to Todeschini et al. (2020) [8] with slight modifications. Briefly, the pH of standards or samples was adjusted to pH 11 with 0.2 M or 6 M NaOH. DMA, TMA, and TMAO contents were analyzed using headspace (HS) gas chromatography (GC) equipped with a nitrogen–phosphorus detector (NPD). Standards or samples were equilibrated for 10 min at 90 °C in a 7697A headspace sampler (Agilent Technologies, Santa Clara, CA, USA) before being injected (1 mL) into a 7890B GC (Agilent Technologies, Santa Clara, CA, USA) equipped with an NPD. Injection was conducted in split mode using a 1:5 ratio and an injector temperature of 250 °C. The loop and transfer line were at 110 °C and 115 °C, respectively. Separation of volatile amines was performed on a capillary CP-Volamine column (30 m length × 0.32 mm id) (Agilent Technologies, Santa Clara, CA, USA). Helium was the carrier gas (3 mL/min). The detector temperature was at 300 °C. GC oven temperature was increased from 50 °C (temperature maintained for 4 min) to 200 °C at 25 °C/min. Calibration curves of known amounts of DMA (from 1 ppm to 20 ppm), TMA (from 1 ppm to 10 ppm), and TMAO (from 10 ppm to 100 ppm) standards were generated. Open Lab CDS EZChrom Software version A.03.02.023 (Agilent Technologies, Santa Clara, CA, USA) was used to process the data.

*Most Potent Odor-Active Compounds*

Extraction of Most Potent Odor-Active Compounds

The extraction procedure was conducted according to Tremblay et al. (2020) [15]. Succinctly, 45 mL liquid samples were absorbed by cotton balls. The cotton balls were then placed into glass tubes connected to an air filtration system for 1 h at room temperature to collect the volatile compounds on a divinylbenzene column (HayeSep^®^ Q 80/100, Bandera, TX, USA). Volatile compounds were recovered with 150 µL of an elution solvent containing dichloromethane and nonyl acetate (internal standard, 3.71 × 10^−5^ M).

Determination of Most Potent Odor-Active Compounds

The most potent odor-active compound contents were determined using a gas chromatography–mass spectrometry (GC–MS) system consisting of a 7890B GC (Agilent Technologies, Santa Clara, CA, USA) equipped with a 5977B mass selective detector (MSD) with a high-efficiency source (Agilent Technologies, Santa Clara, CA, USA). Extracts (1.8 µL) were analyzed according to Tremblay et al. (2020) [15]. Briefly, they were injected in splitless mode into a capillary column (DB-5MS Ultra Inert, 30 m length × 250 µm id, 1 µm thickness) (Agilent Technologies, Santa Clara, CA, USA). Helium was the carrier gas (1.3 mL/min). The GC oven temperature was first increased from 35 °C (temperature maintained for 1.46 min) to 47 °C at 6 °C/min and then increased to 250 °C (temperature maintained for 3 min) at 10 °C/min. The MSD source and the MSD quad were at 230 °C and 150 °C, respectively. The MSD was operated at an ionization energy of 70 eV in scan mode with a scan rate of 6.1 scans/min and an *m*/*z* range scanned between 30 and 250 atomic mass units (a.m.u.). Volatile compounds were identified by matching their mass spectra with those gathered in the NIST 17.L mass spectral database (National Institute of Standards and Technology, Gaithersburg, MD, USA). Since the HMH powder used in this study was from the same lot as Todeschini et al. (2020) [8], the same compounds were considered as being the most potent odor-active ones of HMH, and the area under their peak was considered to assess their abundance. MassHunter Qualitative Analysis software version B.07.00 (Agilent Technologies, Santa Clara, CA, USA) was used to process the data.

b. Sensory Analysis

On the basis of the HS-GC-NPD and GC–MS results, two conditions evidencing the lowest contents in volatile compounds, two conditions evidencing the highest contents, and an intermediate one were considered. Sensory analysis was conducted according to Todeschini et al. (2021) [14]. A panel of 20 judges (15 females and five males between 24 and 52 years old) participated in the sensory analysis session. During this session, 5 mL HMH samples were sniffed at room temperature in crucibles of 40 mL capacity after leaving a 4 °C cold chamber. Samples were placed in a random order, and a three-digit code was ascribed to each of them. The procedure consisted of ranking the five selected conditions from the least to the most odorous. A final *S* score was attributed to each assessed condition (Equation (1)), where *S* was obtained by summing all the rank values *R_i_* given by each panel member [7].

(1) $$S=\sum R_{i}.$$

c. Antioxidant Activity

*Oxygen Radical Absorbance Capacity (ORAC)*

The ORAC test was conducted according to Tremblay et al. (2020) [15]. Briefly, standards and samples were mixed with sodium phosphate buffer prepared at a concentration of 75 mmol/L and a pH of 7.4. Trolox standards were used at concentrations of 12.5, 25, 50, and 100 µM. Hydrolysate samples were assessed at concentrations of 0.5 and 1 mg/mL of peptides. First, 25 µL of standards or samples were transferred into a 96-well black microplate (GreinerBio-One GmbH, Frickenhausen, Germany) and mixed with 150 µL of a fluorescein solution at 0.4 µM. The microplate was preincubated for 30 min at 37 °C before the addition of 50 µL of AAPH to initiate the oxidation reaction. The fluorescence was measured using a Synergy H1 microplate reader (BioTek Instruments, Winooski, VT, USA) every 1 min for 90 min at 37 °C at an excitation wavelength of 485 nm and an emission one of 538 nm. Gen5 software version 2.07.17 (BioTek Instruments, Winooski, VT, USA) was used to process the data. Results were expressed as micromoles of Trolox equivalent per gram of peptides (µmol TE/g).

*DPPH Radical-Scavenging Capacity*

The DPPH radical-scavenging capacity was assessed according to Tremblay et al. (2020) [15] with slight modifications. Briefly, standards and samples were prepared, respectively, in ethanol and ultrapure water. Trolox standards were used at concentrations ranging from 6.25 to 200 µM. Hydrolysate samples were assessed at concentrations of 0.5 and 1 mg/mL of peptides. Then, 50 µL of standards or samples were transferred into a 96-well microplate (VWR, Montréal, QC, Canada) and mixed with 250 µL of DPPH solution at 90 µM prepared in ethanol. The microplate was incubated at 30 °C for 1 h before the absorbance was measured at 517 nm using a microplate spectrophotometer (xMark^TM^, Bio-Rad Laboratories, Inc., Hercules, CA, USA). Ethanol was used as a blank. The radical-scavenging capacity of the samples was determined according to Equation (2).

(2) $$Scavenging\;capacity\;\left(\%\right)=\frac{\left(A_{control}-A_{sample}\right)}{A_{control}}\times 100$$

where *A_control_* and *A_sample_* refer, respectively, to the absorbance of all reagents without the samples and the absorbance of the sample. MPM 6 software version 6.3 (Bio-Rad Laboratories, Inc., Hercules, CA, USA) was used to process the data.

d. Statistical Analyses

Statistical analyses were conducted using SAS software version 9.4 for windows (SAS Institute Inc, Cary, NC, USA). HS-GC-NPD, GC–MS, and antioxidant data were subjected to analyses of variance (ANOVAs), and Tukey tests (α = 0.05 as probability level) were conducted to compare the different conditions. A *t*-test was carried out to compare the antioxidant activity within the same condition. Sensory analysis results were first analyzed using the nonparametric Friedman test to detect any difference between the tested conditions. Then, Wilcoxon test (α = 0.05 as probability level) was used to compare the conditions two by two to identify those different from the others.

## 3. Results and Discussion

### 3.1. Volatile Compound Analyses

#### 3.1.1. DMA, TMA, and TMAO

a. Effects of a 15 min Stirring

The DMA, TMA, and TMAO contents of HMH solutions after the 15 min stirring are shown in Table 1. Firstly, the detected content of DMA was significantly higher in the 15 min stirred HMH solution at pH 7 in comparison with that at pH 11 alkalized with KOH (*p* < 0.05), while the detected content of DMA in the HMH solution at pH 11 alkalized with NaOH was not significantly lower than at pH 7 (*p* > 0.05). These results were not consistent with the ability of alkaline conditions to enhance the availability of DMA and, thus, to result in higher detected content [16]. Indeed, complex matrices, such as HMH, involve various compounds such as peptides, amino acids, lipids, and nucleic acids. Among these compounds, peptides, amino acids, and nucleic acids are able to interact with DMA through the establishment of electrostatic interactions. At pH 11, DMA was present under a neutral form (pKa ≈ 10.70) [17]. This would have decreased its propensity to electrostatically interact with other compounds and would have resulted in higher detected content, independently of the use of NaOH or KOH for the alkalization procedure. To explain such a discrepancy, it could be supposed that a certain part of the DMA content would have been lost during the 15 min stirring at pH 11 due to its highly volatile state. However, this explanation would not be in agreement with the study of Todeschini et al. (2021) [14] in which it was reported that an overnight stirring at pH 10 did not lead to any loss of DMA. The fact that, in this study, the stirring procedure was conducted at pH 11 and not at pH 10 may explain such differences. In any case, even by assuming that the hypothesis regarding the potential loss of DMA could be involved, it would not be sufficient to totally account for such results. This led to consider a second hypothesis involving the occurrence of a potential trapping of DMA into peptide chains due to nonspecific binding at pH 11. In fact, both charge and peptide conformation are impacted by pH change and, as a consequence, the retention ability of volatile compounds, such as DMA, is also impacted [18]. The retention of volatile compounds by non-volatile ones can result from specific or nonspecific binding. Specific binding denotes that interactions including hydrophobic and electrostatic interactions, hydrogen bonds, and Van der Waals forces, in addition to covalent chemical ones, can be involved [19]. Nonspecific binding is more related to the propensity of non-volatile compounds, such as peptides, to trap volatile ones into, for example, hydrophobic pockets, resulting from intramolecular interactions, or into bigger structures resulting from peptide aggregation, for example [19]. Since, at pH 11, DMA was more released from electrostatic interactions and the expected pH effect was not observed, it could be supposed that a part of the DMA content would be involved in nonspecific binding to peptides rather than specific binding such as electrostatic interactions. Since DMA is a small molecule (<60 Da), the hypothesis involving its entrapment into peptide chains would be even more plausible. In addition, at pH 11, the N-term of peptides (pKa ≈ 9.8), as well as of basic amino acids such as lysine (pKa ≈ 10.5) and histidine (pKa ≈ 6.0), was mainly present under a neutral form [14], leading to a decrease in the repulsive electrostatic forces between peptide chains and, thus, resulting in their aggregation and possibly their precipitation. Under these conditions, DMA could be easily trapped, which would corroborate the aforementioned hypothesis. Nonetheless, no precipitation was observed by simple observation of the HMH solutions at pH 11. Moreover, the alkalization procedure was performed either with NaOH or with KOH, both being alkaline salts. These species modified not only the pH but also the ionic strength. Similarly to pH, ionic strength is an important factor impacting the availability of volatile compounds and, at a high concentration of salts, a “salting in” effect has been reported [19]. This means that, at high ionic strength, the promoted interactions between peptides due to the lower availability of water would, at the same time, enhance the retention of volatile compounds [19]. This would be in agreement with the hypothesis formulated previously regarding the retention of a certain part of the DMA content. However, less than 1% (*w/v*) salt was added. This may not be sufficient to totally consider a potential “salting in” effect due to the increase in the ionic strength. Concerning TMA, the 15 min stirred HMH solutions at pH 7 and pH 11 evidenced the same content (*p* > 0.05). Similarly to what was observed with the DMA content, these results were not in line with the ability of alkaline conditions to improve the availability of TMA [16]. Indeed, as DMA, TMA can electrostatically interact with charged compounds such as peptides, amino acids, and nucleic acids. However, under alkaline conditions, TMA was mainly present under a neutral form (pKa ≈ 9.80) [17]. Therefore, a higher detected content in TMA would have been expected for HMH solutions at pH 11, independently of the use of NaOH or KOH for the alkalization procedure. The same hypotheses formulated previously for DMA could be considered for TMA. Lastly, no TMAO was detected in the HMH solutions. This is not in agreement with previous studies evidencing the presence of TMAO in HMH [8,14]. However, TMAO is a compound known to undergo breakdown reactions over time through bacterial or enzymatic actions, even during frozen storage [20]. Therefore, TMAO could have been decomposed during the frozen storage period before the treatments were conducted. Moreover, the fact that, in the present study, higher DMA and TMA contents were observed, in comparison with previous studies dealing with the same lot of HMH [8,14], would give credit to this hypothesis. Interestingly, both HMH solutions alkalized at pH 11 evidenced similar DMA and TMA contents independently of the use of NaOH and KOH. This suggests that NaOH and KOH would have, at that stage, the same effect. In fact, these bases only differ by the monovalent cation they release; the dissociation of NaOH leads to the generation of the Na^+^ cation, while that of KOH leads to the generation of the K^+^ cation. Interestingly, some studies already dealt with the impacts of Na^+^ and K^+^ and noticed that these cations brought about the same effects on volatile compounds, corroborating the results obtained [19,21]. The fact that Na^+^ and K^+^ were reported to evidence the same effects would be related to their very close ranking in the Hofmeister series [19]. The Hofmeister series refers to a classification of ionic species based on their ability to stabilize amino-acid-containing materials in solution [19]. Since amino-acid-containing materials have a huge ability to bind volatile compounds, their stability plays an important role in the availability of volatile compounds [18]. As a consequence, the behavior of volatile compounds is closely related to that of amino-acid-containing materials.

b. Effects of a Single 30 min Deaerator Treatment

The effect of a single 30 min deaerator treatment on the DMA, TMA, and TMAO contents of HMH solutions at pH 7 and pH 11 was assessed (Table 1). Neither the DMA content nor the TMA content of the HMH solution at pH 7 (pH 7 + D30) was impacted by the 30 min deaerator treatment (*p* > 0.05). These results are in line with previous studies reporting that deaerator treatment at neutral pH had no effect on these compounds due to their high propensity to be involved in electrostatic interactions hampering their removal [8,14]. Indeed, on the basis of their pKa values of 10.70 and 9.80, respectively [17], DMA and TMA evidenced a positive charge at pH 7. Consequently, they were able to interact electrostatically with negatively charged compounds such as the C-term of peptides (pKa ≈ 2.1) or the side-chains of aspartic and glutamic acids (pKa ≈ 4.0) in both their bound and their free states [14]. In addition, HMH contains nucleic acids carrying a negative charge, and both DMA and TMA could be involved in interactions with these compounds. Therefore, this seems to demonstrate that the deaerator was not effective to break weak interactions such as electrostatic ones. Regarding the application of a 30 min deaerator treatment on HMH solutions at pH 11 previously alkalized with either NaOH (pH 11 NaOH + D30) or KOH (pH 11 KOH + D30), the detected DMA and TMA contents decreased in both cases (*p* < 0.05). This is in line with the ability of a deaerator to remove gas and, at the same time, to take part in the removal of odorous compounds due to their volatile state. More precisely, in comparison with the 15 min stirred HMH solution alkalized with NaOH, the pH 11 NaOH + D30 solution evidenced decreases in DMA and TMA of 32% and 50%, respectively, while the pH 11 KOH + D30 HMH solution showed decreases in DMA and TMA of 35% and 68%, respectively, compared to the 15 min stirred HMH solution alkalized with KOH. In that case, the high performance of the deaerator in decreasing the DMA and TMA contents at pH 11 could be explained by their lower involvement in electrostatic interactions with the aforementioned compounds since both DMA and TMA were mainly present under a neutral form at pH 11, facilitating their removal by the deaerator. In addition, on the basis of what was hypothesized before regarding the potential trapping of DMA and TMA into peptide chains, the fact that the deaerator treatment was able to decrease their contents at pH 11 would corroborate the occurrence of this phenomenon. Indeed, since the deaerator was able to decrease the DMA and TMA contents, this may indicate that these two compounds were still present in HMH solutions at pH 11; otherwise, the deaerator would not have been effective in bringing about their further removal. This may also indicate that a weak interaction was not necessarily involved at pH 11 between DMA and TMA and the other constituents of HMH, in contrast to what was observed at pH 7, since the deaerator was ineffective in removing these two compounds under neutral conditions. Interestingly, in comparison with the study of Todeschini et al. (2021) [14], in which they conducted 30 min deaerator treatments on HMH solutions at pH 10, deaerator treatments at pH 11 allowed an additional decrease in DMA. This could be related to the fact that, at pH 11, DMA (pKa ≈ 10.70) is no longer charged, unlike at pH 10, which would facilitate its removal. However, Todeschini et al. (2021) [14] reported, in these conditions, a decrease of 80% in the TMA content. In the present study, the decrease in the TMA content was slightly lower. On the whole, there was no significant difference in terms of DMA and TMA removal between deaerator treatments conducted at pH 10 and at pH 11. Accordingly, it would not be totally justified to perform further experiments at pH value of 11. Moreover, statistical analyses revealed no significant difference between the pH 11 NaOH + D30 and pH 11 KOH + D30 conditions. Therefore, this suggests that NaOH could be substituted by KOH without affecting the deaerator performance.

c. Effects of a Combination of 15 min Deaerator Treatments

In addition to single 30 min deaerator treatments, a combination of 15 min deaerator treatments at both pH 7 and pH 11 was assessed (Table 1). No difference was observed between the DMA and TMA contents of the 15 min stirred HMH solution at pH 7 and those treated by the deaerator for 15 min (pH 7 + D15) (*p* > 0.05). These results are in agreement with what was already observed regarding the pH 7 + D30 condition. While the pH 7 + D15 HMH solution underwent a further alkalization step to pH 11 with NaOH or KOH, the DMA content significantly decreased (*p* < 0.05), whereas the TMA one remained the same (*p* > 0.05). This seems to corroborate the previous explanation. In addition, this confirmed that the DMA and TMA contents were not affected by the base used for the alkalization procedure. Lastly, when a 15 min deaerator treatment was conducted on the pH 7 + D15 + pH 11 NaOH HMH solution (pH 7 + D15 + pH 11 NaOH + D15), a drop in the DMA and TMA contents of respectively 36% and 68% was observed (*p* < 0.05). The same trend was observed for the pH 7 + D15 + pH 11 KOH + D15 condition. In addition, the DMA and TMA contents of both pH 7 + D15 + pH 11 NaOH + D15 and pH 7 + D15 + pH 11 KOH + D15 conditions were the same (*p* > 0.05). This confirmed that the base used had no effect on the deaerator performance, and that the application of a deaerator treatment under alkaline conditions was an effective strategy to decrease the DMA and TMA contents. 

#### 3.1.2. Most Potent Odor-Active Compounds

a. Effects of 15 min Stirring

The most potent odor-active compound contents of HMH solutions after the 15 min stirring step are shown in Table 2. First, the abundance of these compounds was globally lower in the HMH solutions alkalized at pH 11 with NaOH or KOH. More precisely, the contents of 3-methylbutanal, 1-methyl-1*H*-tetrazole, 2,3-pentanedione, pentanal, (*Z*)-4-heptenal, methional, benzaldehyde, (*E*,*E*)-2,4-heptadienal, octanal, 2-nonanone, and (*E*,*Z*)-2,6-nonadienal were lower in the pH 11 HMH solution alkalized with NaOH in comparison with the pH 7 solution (*p* < 0.05). In addition to these compounds, the pH 11 HMH solution alkalized with KOH evidenced a significant decrease in the abundance of 2-methylbutanal and hexanal compared to the pH 7 solution (*p* < 0.05). As mentioned for DMA and TMA, this difference in terms of volatile compound contents was consistent with the ability of pH to impact their availability [18]. However, in contrast to DMA and TMA, the global lower contents obtained for the HMH solutions at pH 11 were in line with what has already been observed in the literature [8,14]. Recently, Todeschini et al. (2021) [14] suggested that the observed decrease in the most potent odor-active compound contents of HMH solution observed under alkaline conditions could be related to a drop in their volatile state. This would mean that the targeted compounds would still be present in the HMH solutions at basic pH. However, they would no longer be available for detection, probably involved in interactions with other compounds of HMH. Moreover, except for hexanal, the two pH 11 HMH solutions had a similar composition.

b. Effects of a Single 30 min Deaerator Treatment

The most potent odor-active compound contents of HMH solutions produced over a single 30 min deaerator treatment are shown in Table 2. Concerning the deaerator treatment conducted on HMH solution at pH 7 (pH 7 + D30), a decrease from around 30% to 100% was observed for the following compounds in comparison with the pH 7 condition after the 15 min stirring (*p* < 0.05): 3-methylbutanal, 2-methylbutanal, 1-methyl-1*H*-tetrazole, 2,3-pentanedione, pentanal, (*Z*)-4-heptenal, benzaldehyde, (*Z*)-6-octen-2-one, octanal, 2-nonanone, and (*E*,*Z*)-2,6-nonadienal. The observed decrease was consistent with the ability of a deaerator to remove gas from a matrix and, thus, to bring about the removal of the odorous compounds due to their volatile state. The observed decrease was of the same order as those mentioned by Todeschini et al. (2021) [14] for similar treatment conditions. Then, regarding the application of a 30 min deaerator treatment on HMH solution alkalized at pH 11 with NaOH (pH 11 NaOH + D30), no significant difference was observed in comparison with the HMH solution stirred for 15 min (*p* > 0.05). Regarding the application of a 30 min deaerator treatment on a HMH solution alkalized to pH 11 with KOH (pH 11 KOH + D30), only 2-nonanone content dropped significantly in comparison with the HMH solution alkalized at pH 11 with KOH, which was just stirred for 15 min (*p* < 0.05). Interestingly, the most potent odor-active compound contents of pH 11 NaOH + D30 and pH 11 KOH + D30 were quite similar. On the one hand, this would corroborate what was observed previously concerning the similar composition of the HMH solutions alkalized to pH 11 with either NaOH or KOH. On the other hand, this would indicate that the type of bases used did not impact the performance of the deaerator, even for the most potent odor-active compounds. The ineffectiveness of the deaerator at pH 11 would give credit to the fact that, under alkaline conditions, the targeted compounds would be involved in bindings with other constituents of HMH. Interestingly, the ineffectiveness of the deaerator to remove the most potent odor-active compounds of HMH under alkaline conditions was consistent with Todeschini et al. (2020, 2021) [8,14]. Moreover, as observed previously at pH 11, the deaerator was still effective to reduce the DMA and TMA contents in spite of their potential trapping into peptide chains. Thus, this seems to indicate that two different mechanisms would be involved for the most potent odor-active compounds and for DMA and TMA under alkaline conditions. To better characterize the mechanism responsible for the observed decrease in the abundance of the most potent odor-active compounds of HMH, an additional acidification to pH 7 was performed following the 30 min deaerator treatments on pH 11 NaOH (pH 11 NaOH + D30 + pH 7) and pH 11 KOH HMH (pH 11 KOH + D30 + pH 7) solutions (Table 2). Interestingly, this led to the significant increase in 2-methylbutanal, 2,3-pentanedione, pentanal, (*Z*)-4-heptenal, methional, benzaldehyde, octanal, and (*E*,*Z*)-2,6-nonadienal contents of the pH 11 NaOH + D30 + pH 7 solution in comparison with the pH 11 NaOH + D30 solution (*p* < 0.05). A similar trend was observed for the pH 11 KOH + D30 + pH 7 solution, which evidenced a further increase in the 2,3-pentanedione, methional, benzaldehyde, (*E*,*E*)-2,4-heptadienal, and (*E*,*Z*)-2,6-nonadienal contents compared to the pH 11 KOH + D30 solution (*p* < 0.05). This suggests that the most potent odor-active compounds of HMH would be involved in noncovalent binding at basic pH and would be released from these interactions under neutral conditions. On the one hand, the potential involvement in noncovalent binding of the targeted compounds at alkaline pH values would confirm the reason why their abundances were lower under these conditions. In addition, this would give credit to the ineffectiveness of the deaerator to break weak interactions. At that stage, the involvement of covalent binding was definitely excluded, as this type of binding is irreversible. On the other hand, the better availability of these compounds at neutral pH would, finally, explain the reason why the deaerator was more effective in their removal under these conditions.

c. Effects of a Combination of 15 min Deaerator Treatments

The 15 min deaerator treatment conducted on HMH solution at pH 7 (pH 7 + D15) allowed a decrease in the abundance of 2-methylbutanal, 2,3-pentanedione, and (*Z*)-4-heptenal in comparison with the pH 7 solution stirred for 15 min (*p* < 0.05) (Table 2). Interestingly, the pH 7 + D30 condition evidenced lower contents of the targeted compounds. This may indicate that the treatment duration had a significant effect on the removal rate of volatile compounds and on the process performance. Nonetheless, the importance of the treatment duration was not perceived for DMA and TMA since the 15 min deaerator treatment removed as much DMA and TMA as the 30 min one. The fact that DMA and TMA are smaller molecules would make their removal by the deaerator quicker. In addition, while the pH 7 + D15 HMH solution was alkalized to pH 11 with NaOH (pH 7 +D15 + pH 11 NaOH), a further significant drop in the 3-methylbutanal, 1-methyl-1*H*-tetrazole, 2,3-pentanedione, pentanal, hexanal, (*Z*)-4-heptenal, heptanal, methional, benzaldehyde, (*E*,*E*)-2,4-heptadienal, octanal, and (*E*,*Z*)-2,6-nonadienal contents was observed (*p* < 0.05). All these compounds followed a similar decrease for the pH 7 + D15 HMH solution alkalized with KOH (pH 7 + D15 + pH 11 KOH) (*p* < 0.05) except for hexanal (*p* > 0.05). This was consistent with what was observed previously regarding the global lower volatile compound contents of alkalized solutions, independently of the use of NaOH or KOH, in comparison with the neutral solutions. Interestingly, in comparison with the pH 11 HMH solution alkalized with NaOH and stirred for 15 min, the pH 7 + D15 + pH 11 NaOH solution evidenced significant lower contents of 3-methylbutanal, 2-methylbutanal, hexanal, and 2-nonanone (*p* < 0.05). For the pH 7 + D15 + pH 11 KOH condition, a downward trend was only observed regarding the 2-nonanone content in comparison with the 15 min stirred HMH solution alkalized at pH 11 with KOH. As observed previously, the deaerator appears to be more effective at pH 7 while the alkalization of the HMH solution also led to a reduction in the abundance of the targeted compounds. Thus, in the case of the pH 7 + D15 + pH 11 NaOH, more specifically, the higher decrease in the volatile compound contents would probably be related to the combination of the high performance of both the deaerator treatment at pH 7 and the further alkalization. This high performance of the combination of both alkaline pH and deaerator treatment at pH 7 was even visible when comparing, on the one hand, the pH 7 + D30 and pH 7 + D15 + pH 11 NaOH conditions and, on the other hand, the pH 7 + D30 and pH 7 + D15 + pH 11 KOH conditions. Although a single 15 min deaerator treatment at pH 7 was less optimal than a single 30 min one for the removal of the targeted compounds, conducting a further alkalization step at pH 11 enhanced its performance. Lastly, while the pH 7 + D15 + pH 11 NaOH and the pH 7 + D15 + pH 11 KOH conditions followed a 15 min deaerator treatment (pH 7 + D15 + pH 11 NaOH + D15; pH 7 + D15 + pH 11 KOH + D15), no further decrease in the targeted compounds was observed in both cases (*p* > 0.05). This corroborated the ineffectiveness of the deaerator to reduce the most potent odor-active compound contents of HMH under alkaline conditions due to their involvement in weak interactions with other components of HMH.

#### 3.1.3. General Discussion on Volatile Compounds

Results demonstrated that the applications of pH 7 + D15 + pH 11 NaOH + D15, pH 7 + D15 + pH 11 KOH + D15, pH 11 NaOH + D30, and pH 11 KOH + D30 were the most effective to reduce the DMA and TMA contents. Regarding the most potent odor-active compounds of HMH, the application of pH 7 + D15 + pH 11 NaOH and pH 7 + D15 + pH 11 KOH could be considered as optimal to decrease their content. Nonetheless, to guarantee a high deodorization degree of HMH, both the amine compounds and the most potent odor-active ones should be taken into account. Consequently, the optimal conditions appear to be pH 7 + D15 + pH 11 NaOH + D15 and pH 7 + D15 + pH 11 KOH + D15. Moreover, as the results showed that these compounds were not impacted by the base used, the pH 7 + D15 + pH 11 KOH + D15 condition could definitely be considered as the best avenue for HMH deodorization.

In terms of TMA and DMA removal, the pH 7 + D15 + pH 11 KOH + D15 condition led to a decrease in the two amines by 70%. In comparison with other deodorization strategies, this is the first time that a deodorization method applied to a marine resource has allowed such a decrease in the DMA and TMA contents simultaneously, since other strategies mainly focused on TMA removal. The treatment developed in the case of TMA removal seems to be of higher performance than reported in the literature. Indeed, Park et al. (2020) [10] observed that lactic acid bacteria were able to remove up to 62% of the TMA content of spoiled fish, while Jo et al. (2021) [11] instead identified the use of such microorganisms as a strategy to prevent the formation of TMA in ribbonfish fillets. Similarly, the use of tea polyphenols in clam hydrolysate was identified as a strategy to prevent the formation of TMA [7]. Lastly, using a physical method based on activated carbon adsorption, Boraphech and Thiravetyan (2015) reached a TMA removal rate similar to that obtained in this study. However, in that case, experiments were conducted on a pure solution of TMA [13]. Therefore, this would demonstrate the high potential of the deodorization method developed in this study to be applied to complex matrices. Moreover, in terms of most potent odor-active compound removal, compared with other deodorization strategies reported in the literature, the pH 7 + D15 + pH 11 KOH + D15 condition appeared to be of higher performance again. In fact, the use of tea polyphenols only allowed decreases of, respectively, 19% and 25% in the benzaldehyde and heptanal contents of a clam hydrolysate [7], while the application of the pH 7 + D15 + pH 11 KOH + D15 condition removed, respectively, 76% and 46% of the benzaldehyde and heptanal contents of HMH. Song et al. (2018) [12] noticed that tea polyphenols had almost no effect on the benzaldehyde content of fish oil, whereas they reached a removal rate of 27% for the 2-nonanone content. In this study, the pH 7 + D15 + pH 11 KOH + D15 reduced the 2-nonanone content by 83%. Regarding physical methods such as the use of activated carbon, Li et al. (2020) [22] removed, respectively, 31% and 36% of the benzaldehyde and octanal contents of a fish hydrolysate, while, in this study, 72% of the octanal was removed. Using activated carbon, Chen et al. (2016) [7] managed to remove 58% of the pentanal in a clam hydrolysate, while, in this study, 70% of this compound was removed. using another adsorption method based on the use of zeolite, Güner et al. (2019) [23] removed 60% of the (*E*,*E*)-2,4-heptadienal in fish oil, while the pH 7 + D15 + pH 11 KOH + D15 condition managed to remove almost 100% of the (*E*,*E*)-2,4-heptadienal content of HMH. Therefore, these results definitely showed the high performance of the combination of deaerator treatments in the deodorization of complex matrices.

### 3.2. Sensory Analysis

Sensory analysis was performed to assess if the results obtained using analytical procedures could be corroborated by human perception. The five conditions subjected to sensory analysis were the following: pH 7 after 15 min stirring, pH 7 + D30, pH 11 KOH after 15 min stirring, pH 11 KOH + D30, and pH 7 + D15 + pH 11 KOH + D15. Results indicated that the pH 7 after 15 min stirring and pH 7 + D30 conditions were perceived as being the most odorous in comparison with the other assessed conditions (*p* < 0.05) since they both obtained the highest S score (Table 3). Regarding the pH 7 after 15 min stirring condition, this was in line with both HS-GC-NPD and GC–MS results. Indeed, this condition evidenced the highest content in the most potent odor-active compounds, as well as the highest content of TMA and DMA. On the one hand, among the most potent odor-active compounds of HMH, pentanal, hexanal, (*Z*)-4-heptenal, heptanal, (*E*,*E*)-2,4-heptadienal, and octanal are associated with unappealing odor descriptors. Indeed, hexanal, (*Z*)-4-heptenal, heptanal, (*E*,*E*)-2,4-heptadienal, and octanal are responsible for fishy smell, while pentanal is characterized by a pungent odor [24]. On the other hand, both TMA and DMA are ascribed to fishy and ammoniacal odor descriptors [9,24]. Therefore, this may explain the reason why the highest S score value was ascribed to the pH 7 after 15 min stirring condition. Surprisingly, no discrimination was observed between the pH 7 after 15 min stirring and pH 7 + D30 conditions (*p* > 0.05). This was not totally in accordance with GC–MS results indicating a lower content of the most potent odor-active compounds of HMH after the 30 min deaerator treatment. However, the fact that the pH 7 + D30 and the pH 7 after 15 min stirring conditions showed a similar content of TMA and DMA could explain the reason why they were identified as being the two most odorous ones. All the conditions at pH 11 were perceived as being the least odorous ones (*p* < 0.05). These results were in agreement with the GC–MS, results indicating a lower content of the most potent odor-active compounds at pH 11 than at pH 7 due to their involvement in weak interactions. Interestingly, among the most potent odor-active compounds whose contents were lower at pH 11, (*E*,*E*)-2,4-heptadienal and (*E*,*Z*)-2,6-nonadienal were reported to evidence synergistic effects [15]. In fact, while these compounds are present together, (*E*,*Z*)-2,6-nonadienal, initially ascribed to a pleasant cucumber-like smell [25], promotes the fishy off-flavors of (*E*,*E*)-2,4-heptadienal [26]. Synergistic effects between (*Z*)-4-heptenal and other odorous compounds have also been reported. Indeed, (*Z*)-4-heptenal has the capacity to give potency to the odor of other compounds rather than making a huge contribution to the global odor of a matrix on its own [25,26]. Therefore, the lower content of especially (*E*,*E*)-2,4-heptadienal, (*E*,*Z*)-2,6-nonadienal, and (*Z*)-4-heptenal in the conditions at pH 11 could also have accounted for their lowest S scores. Surprisingly, panel members did not discriminate between the conditions at pH 11 (*p* > 0.05). Since all the conditions assessed at pH 11 showed a similar content of the most potent odor-active compounds, this was quite logical. However, the pH 11 KOH + D30 and pH 7 + D15 + pH 11 KOH + D15 conditions had a lower content of both TMA and DMA in comparison with the pH 11 KOH after 15 min stirring condition, and this should have been perceived. Nonetheless, the fact that the conditions at pH 11 evidenced a high performance in decreasing the availability of the most potent odor-active compounds could account for the lowest contribution of TMA and DMA to the resulting odor of HMH. This could be corroborated by the fact that (*Z*)-4-heptenal could give potency to the odor of other compounds, whereas its content of was lower at pH 11.

As a whole, the sensory analysis corroborated the results obtained using analytical procedures. In addition, it appears that the tendency of basic conditions to reduce the availability of the most potent odor-active compounds of HMH was prevalent in its olfactory perception. In a deodorization context, the optimal condition would be to reduce the content of the targeted compounds by removing them totally instead of just making them unavailable. This would be even more necessary for a product that could be consumed such as HMH, which is already used as a food supplement, to avoid the uncontrolled and undesired release of odorous compounds. Indeed, consumption of food is a complex process composed of various steps [27]. The first is mastication, bringing about the decomposition of the food product into fragments. At that stage, a release of volatile compounds could happen due to changes in food structure caused by the mastication process and the contribution of saliva present in the mouth [27]. Since saliva has a normal pH value ranging from 6.2 to 7.4 [28] and since it was shown that the most potent odor-active compounds tended to be more released under neutral conditions, the consumption of deodorized HMH at pH 11 could be potentially associated with the release of these compounds into the mouth cavity. For this reason, the application of pH 7 + D15 + pH 11 KOH + D15 appears to be definitely the best option since it allows an initial removal of the most potent odor-active compounds of HMH using a deaerator. Then, while the food product goes deeper into the human organism, the change in pH conditions would probably also lead to the potential release of volatile compounds. As a consequence, for further studies, it would be of particular interest to deepen the knowledge regarding the potential release of the targeted compounds during consumption. This could be achieved through either continuous in vivo methods such as atmospheric pressure chemical ionisation mass spectrometry (API-MS) and proton transfer reaction mass spectrometry (PTR-MS) or discontinuous in vivo ones such as a retronasal aroma-trapping device (RATD) [27].

### 3.3. Antioxidant Activity

#### 3.3.1. ORAC

**Figure 2:**
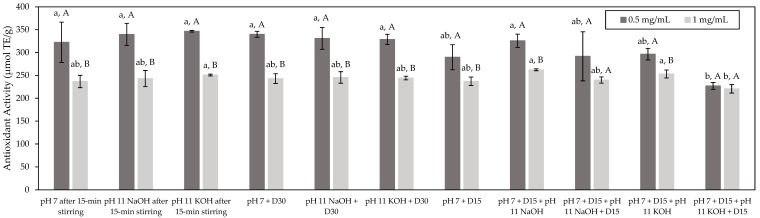
Antioxidant activity of the HMH solutions (µmol TE/g) corresponding to the different tested conditions and determined by the ORAC test (mean ± standard deviation). Values with different lowercase letters (a,b) within the same concentration are significantly different *p* < 0.05 (Tukey test); values with different uppercase letters (A,B) within the same condition are significantly different *p* < 0.05 (*t*-test).

The antioxidant activity of fish hydrolysates can be ascribed to different mechanisms. For this reason, it is of importance to use at least two different tests based on different mechanisms to gain better insight [29]. Accordingly, the ORAC test was first used to assess the antioxidant potential of the studied HMH, as well as the impact of the different conditions. The ORAC test is based on the prevention of the degradation of a fluorescein probe by the scavenging of peroxyl radicals originating from AAPH. This scavenging results in a limited loss of florescence [15]. Results (Figure 2) showed that, globally, all the tested conditions evidenced a similar antioxidant activity for a given concentration (*p* > 0.05). Indeed, at a concentration of 0.5 mg/mL of peptides, all the tested conditions presented an average antioxidant activity of 310 µmol TE/g, while, at a concentration of 1.0 mg/mL of peptides, an average antioxidant activity of 245 µmol TE/g was obtained. Statistical analyses may suggest that the antioxidant activity of the HMH solution produced in the application of the pH 7 + D15 + pH 11 KOH + D15 condition would be lower than that in the other conditions for the two tested concentrations (*p* < 0.05). However, since the antioxidant activities reported for this condition remained on the same order as those of the other conditions, the detected differences, in that case, could be considered as minor. Globally, these results seem to indicate that neither the application of a deaerator treatment nor the different tested pH values had an impact on the antioxidant potential of HMH independently of the use of NaOH or KOH with regard to the conditions at basic pH. Therefore, this may suggest that it could be possible to deodorize HMH without affecting its bioactive potential. This means that the volatile compounds targeted in this study would evidence no antioxidant activity. In fact, in the literature, volatile compounds described as having antioxidant potential include mainly terpenes and sulfur-containing compounds [30,31,32] and, in the present case, none of the targeted odorous compounds belonged to these groups except for methional, being a sulfur-containing compound. However, the fact that its antioxidant potential was not observed in this study could be explained by the fact that either its abundance was too low, even in the untreated HMH, or potential inhibitors could be present hindering any antioxidant activity. At this stage, it is worth mentioning that the antioxidant properties of fish hydrolysates are mainly ascribed to the bioactive peptides they could contain [33]. Generally, the antioxidant properties of such compounds are influenced by their amino-acid sequence and composition, in addition to their degree of hydrophobicity [34,35]. Since the deaerator treatment had no effect on these parameters, this could explain why all the conditions tested in this study evidenced a similar antioxidant activity. Nonetheless, the fact that pH had no impact was more surprising. Indeed, pH influences the global charge of peptides, and it was reported that basic peptides had greater antioxidant capacity than acidic or neutral ones [34]. The fact that no pH effect was observed in the present case could be due to the discrepancy between the methods used in this study and those reported in the literature to assess the antioxidant properties, since they could be based on different mechanisms. Moreover, for most of the tested conditions, the higher antioxidant activity was obtained at 0.5 rather than 1.0 mg/mL of peptides (*p* > 0.05). This may be explained by the fact that the ORAC assay is known for being highly sensitive and samples should be sufficiently diluted to avoid interferences [36]. Therefore, the lower antioxidant activity observed at 1.0 mg/mL might be due to the potential occurrence of interference due to the higher concentration assessed in that case. Interestingly, in their study, Durand et al. (2019) [2] reported a similar antioxidant capacity of 218.32 ± 8.91 µmol TE/g for an HMH with a composition very close to that studied in the present case. In addition, the ORAC values obtained for the studied HMH were comparable to those of other fish protein hydrolysates such as Pacific hake protein hydrolysates for which ORAC values ranging from 225 to 330 µmol TE/g were observed [37,38]. Other fish protein hydrolysates such as those originating from cod bones or salmon frames were reported in the literature to have higher antioxidant activity, with ORAC values ranging from 435 to 528 µmol TE/g and from 544 to 840 µmol TE/g, respectively [39,40]. Nevertheless, in that case, the studied hydrolysates contained higher contents of purified peptides than the present study, thus explaining such higher ORAC values [39,40]. Interestingly, Durand et al. (2019) [2] reported in a similar manner that the antioxidant potential of HMH could be enhanced almost threefold following a purification step, since they managed to isolate from the initial HMH a fraction having an antioxidant activity of almost 700 µmol TE/g. 

#### 3.3.2. DPPH Radical-Scavenging Capacity

**Figure 3:**
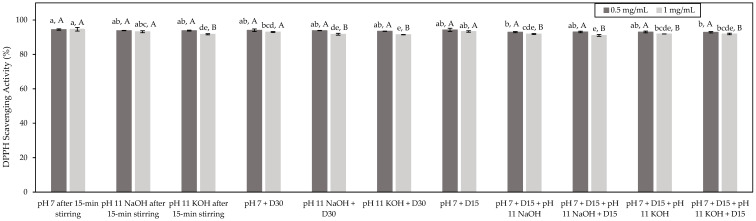
Antioxidant activity of the HMH solutions corresponding to the different tested conditions and determined by the DPPH test (mean ± standard deviation) expressed in DPPH scavenging activity (%). Values with different lowercase letters (a–e) within the same concentration are significantly different *p* < 0.05 (Tukey test); values with different uppercase letters (A,B) within the same condition are significantly different *p* < 0.05 (*t*-test).

The DPPH test was also conducted to assess the antioxidant potential of the studied HMH and the impact of the different conditions. The DPPH test is based on the scavenging of DDPH free radicals by an antioxidant material. This scavenging results in a loss of the absorbance of the DDPH solution [15]. Results expressed as the percentages of DPPH scavenging activity were presented in Figure 3. Globally, all the tested conditions showed a similar DPPH scavenging activity at a tested concentration of 0.5 mg/mL of peptides (*p* > 0.05) with an average DPPH scavenging activity of 94% (Figure 3). Similarly to what was observed for the ORAC test, the DPPH scavenging activity of the pH 7 + D15 + pH 11 KOH + D15 condition was identified as being significantly lower than those of the other conditions by statistical analyses (*p* < 0.05). Nonetheless, as mentioned before, since the DPPH scavenging activity obtained for this condition was on the same order as the other conditions, the detected differences could be perceived as minor. At a tested concentration of 1.0 mg/mL of peptides, all the conditions showed an average DDPH scavenging activity of 92%. Despite the fact that statistical analyses identified the pH 7 after 15 min stirring and the pH 7 + D15 + pH 11 NaOH + D15 condition as being those with the highest and the lowest DPPH scavenging activity (*p* < 0.05), respectively, since the values were all in the same range, the detected differences could be considered as minor. In addition, for most of the tested conditions, the highest DDPH scavenging activity was obtained at a concentration of 0.5 mg/mL of peptides rather than 1.0 mg/mL of peptides (*p* > 0.05). This might be due to the potential occurrence of interference resulting from the presence of pigments in HMH solutions. In fact, the DPPH test is based on the change in absorbance of the DPPH solution; thus, the presence of colored compounds could lead to the underestimation of the antioxidant activity [41]. Since the HMH solutions at 1.0 mg/mL of peptides were more concentrated than those at 0.5 mg/mL of peptides and, as a consequence, their color was more pronounced, this could explain their lower antioxidant activity. It appears from these results that the assessed conditions had no impact on the antioxidant potential of HMH, similarly to what was observed with the ORAC test, and the same explanations could be involved. Interestingly, other fish protein hydrolysates such as catfish protein hydrolysate and cod protein hydrolysate evidenced DPPH scavenging activity equal to 30% and 19%, respectively [42,43]. The literature has also reported DPPH scavenging activity ranging from 52% to almost 80% for unfractionated silver carp protein hydrolysate and silver carp protein hydrolysate, respectively, composed mainly of low-molecular-weight peptides at a concentration of 2 mg/mL [35]. Therefore, in comparison with other fish protein hydrolysates, it seems that the studied HMH showed an important DPPH scavenging activity, even in an unfractionated state.

**Figure 4:**
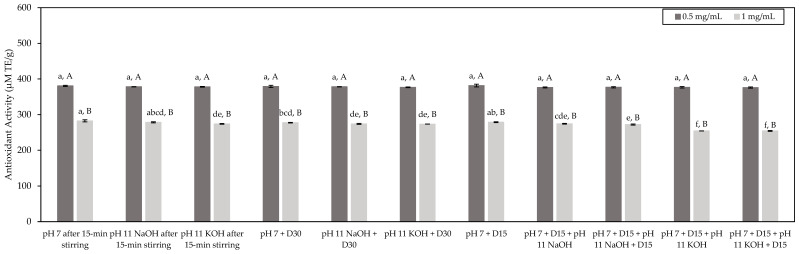
Antioxidant activity of the HMH solutions corresponding to the different tested conditions and determined by the DPPH test (mean ± standard deviation) expressed in µmol TE/g. Values with different lowercase letters (a–f) within the same concentration are significantly different *p* < 0.05 (Tukey test); values with different uppercase letters (A,B) within the same condition are significantly different *p* < 0.05 (*t*-test).

Results were expressed in µmol TE/g, as presented in Figure 4. The same trend as observed before was obtained. Indeed, for a given concentration, all the tested conditions evidenced a similar antioxidant potential with average antioxidant activities of 378 µmol TE/g and 272 µmol TE/g at tested concentrations equal to 0.5 mg/mL and 1.0 mg/mL of peptides, respectively. In addition, for all the test conditions, higher antioxidant activity was obtained at 0.5 mg/mL of peptides rather than 1.0 mg/mL of peptides. Interestingly, the values of antioxidant activity determined by the DPPH test were in the same range as those determined by the ORAC test.

As a whole, results obtained by ORAC and DPPH tests seem to demonstrate the feasibility of deodorizing HMH without affecting its antioxidant properties. Moreover, the values of antioxidant activities obtained in the present study may indicate that HMH could have huge potential as a new natural antioxidant.

## 4. Conclusions

This study aimed to optimize a deodorization method of HMH using a deaerator. To optimally deodorize HMH, the combination of deaerator treatments at neutral and basic pH was demonstrated as being the best avenue according to analytical and sensory procedures. Interestingly, the substitution of NaOH by KOH had no impact on the process performance. This indicates that KOH could definitely be used instead of NaOH while guaranteeing a high process performance. Moreover, results showed that the antioxidant properties of HMH were impacted neither by the deaerator nor by the pH, independently of the base used for alkalization.

Therefore, this demonstrates the feasibility of the method developed in this study to effectively deodorize HMH without affecting its bioactive capacity. To support these promising results, it could be interesting to study the release of volatile compounds during consumption since the pathways involved in the perception of odorous compounds differ when the product is sniffed or consumed. In addition, it could be relevant to deepen the knowledge regarding the bioactive potential of the deodorized HMH by assessing, in particular, its anti-inflammatory and antidiabetic activities, since they were only demonstrated for the raw product that was not deodorized.

## Figures and Tables

**Table 1:** DMA, TMA, and TMAO contents of HMH (in parts per million (ppm)) after the 15 min stirring and after the different treatments (mean ± standard deviation).

	15 min Stirring			Single Deaerator Treatment			Combined Deaerator Treatments				
	pH 7	pH 11 NaOH	pH 11 KOH	pH 7 + D30	pH 11 NaOH + D30	pH 11 KOH + D30	pH 7 + D15	pH 7 + D15 + pH 11 NaOH	pH 7 + D15 + pH 11 NaOH + D15	pH 7 + D15 + pH 11 KOH	pH 7 + D15 + pH11 KOH + D15
DMA	21.22 ± 1.95 ^ab^	16.69 ± 1.08 ^bc^	14.29 ± 0.66 ^cde^	23.32 ± 1.86 ^a^	11.41 ± 1.36 ^def^	9.18 ± 0.76 ^f^	23.44 ± 2.08 ^a^	15.13 ± 0.94 ^cd^	9.65 ± 0.34 ^ef^	12.28 ± 2.78 ^cdef^	7.80 ± 2.11 ^f^
TMA	6.54 ± 0.16 ^a^	7.50 ± 0.60 ^a^	7.38 ± 0.38 ^a^	6.61 ± 0.12 ^a^	3.74 ± 1.60 ^b^	2.38 ± 0.18 ^bc^	6.61 ± 0.40 ^a^	6.99 ± 0.25 ^a^	2.24 ± 0.51 ^bc^	6.19 ± 0.81 ^a^	1.86 ± 0.49 ^c^
TMAO	<2.50 ^a^	<2.50 ^a^	<2.50 ^a^	<2.50 ^a^	<2.50 ^a^	<2.50 ^a^	<2.50 ^a^	<2.50 ^a^	<2.50 ^a^	<2.50 ^a^	<2.50 ^a^

Values within the same row with different letters (a–f) are significantly different *p* < 0.05 (Tukey test).

**Table 2:** Abundance of the most potent odor-active compounds of HMH (×10^7^ arbitrary units (AUs)) after the 15 min stirring and after the different treatments (mean ± standard deviation).

	15 min Stirring			Single Deaerator Treatment					Combined Deaerator Treatments				
	pH 7	pH 11 NaOH	pH 11 KOH	pH 7 + D30	pH 11 NaOH + D30	pH 11 NaOH + D30 + pH 7	pH 11 KOH + D30	pH 11 KOH + D30 + pH 7	pH 7 + D15	pH 7 + D15 + pH 11 NaOH	pH 7 + D15 + pH 11 NaOH + D15	pH 7 + D15 + pH 11 KOH	pH 7 + D15 + pH 11 KOH + D15
3-Methylbutanal	7.72 ± 0.53 ^a^	5.50 ± 0.08 ^bcd^	3.99 ± 0.61 ^de^	4.47 ± 0.30 ^cde^	4.88 ± 0.54 ^bcde^	5.71 ± 0.52 ^bc^	3.76 ± 0.22 ^e^	3.61 ± 0.42 ^e^	6.32 ± 0.65 ^ab^	3.56 ± 0.40 ^e^	3.50 ± 0.70 ^e^	4.62 ± 0.09 ^cde^	3.88 ± 0.88 ^e^
2-Methylbutanal	4.07 ± 0.24 ^a^	3.33 ± 0.09 ^ab^	2.24 ± 0.44 ^bcde^	1.35 ± 0.19 ^de^	2.64 ± 0.45 ^bcd^	2.96 ± 0.74 ^abc^	1.93 ± 0.43 ^cde^	1.41 ± 0.32 ^de^	2.47 ± 0.89 ^bcde^	1.17 ± 0.37 ^e^	1.37 ± 0.33 ^de^	2.22 ± 0.35 ^bcde^	1.56 ± 0.37 ^de^
1-Methyl-1*H*-tetrazole	4.66 ± 1.06 ^a^	0.00 ± 0.00 ^b^	0.00 ± 0.00 ^b^	0.00 ± 0.00 ^b^	0.00 ± 0.00 ^b^	0.00 ± 0.00 ^b^	0.00 ± 0.00 ^b^	0.00 ± 0.00 ^b^	3.14 ± 2.73 ^a^	0.00 ± 0.00 ^b^	0.00 ± 0.00 ^b^	0.00 ± 0.00 ^b^	0.00 ± 0.00 ^b^
2,3-Pentanedione	2.70 ± 0.31 ^a^	0.18 ± 0.07 ^ef^	0.13 ± 0.09 ^f^	1.18 ± 0.29 ^c^	0.071 ± 0.032 ^f^	1.00 ± 0.07 ^cd^	0.069 ± 0.017 ^f^	0.59 ± 0.03 ^de^	2.21 ± 0.33 ^b^	0.066 ± 0.047 ^f^	0.078 ± 0.025 ^f^	0.12 ± 0.02 ^f^	0.077 ± 0.013 ^f^
Pentanal	4.33 ± 0.47 ^a^	2.06 ± 0.15 ^cde^	1.33 ± 0.28 ^de^	2.31 ± 0.52 ^bcd^	1.40 ± 0.20 ^de^	2.71 ± 0.39 ^bc^	1.10 ± 0.07 ^e^	1.77 ± 0.43 ^cde^	3.28 ± 0.69 ^ab^	1.00 ± 0.27 ^e^	1.04 ± 0.27 ^e^	1.82 ± 0.09 ^cde^	1.30 ± 0.42 ^de^
Hexanal	5.66 ± 0.85 ^a^	5.24 ± 0.65 ^a^	2.79 ± 0.40 ^bcd^	5.32 ± 0.64 ^a^	4.07 ± 1.53 ^abc^	4.76 ± 0.33 ^ab^	3.50 ± 0.42 ^abcd^	3.63 ± 0.39 ^abcd^	4.70 ± 0.67 ^ab^	1.77 ± 0.29 ^d^	2.15 ± 0.92 ^cd^	5.30 ± 0.25 ^a^	4.67 ± 1.20 ^ab^
(*Z*)-4-Heptenal	8.80 ± 1.41 ^a^	1.87 ± 0.22 ^de^	1.99 ± 0.41 ^de^	2.57 ± 0.21 ^de^	1.91 ± 0.53 ^de^	5.29 ± 0.22 ^bc^	1.77 ± 0.08 ^de^	3.93 ± 0.83 ^cd^	6.60 ± 1.73 ^b^	1.69 ± 0.33 ^e^	1.57 ± 0.50 ^e^	1.83 ± 0.34 ^de^	1.55 ± 0.45 ^e^
Heptanal	0.83 ± 0.36 ^abc^	0.46 ± 0.05 ^bc^	0.41 ± 0.11 ^bc^	1.04 ± 0.20 ^ab^	0.32 ± 0.14 ^bc^	1.02 ± 0.15 ^ab^	0.34 ± 0.04 ^bc^	0.80 ± 0.25 ^bc^	1.62 ± 0.78 ^a^	0.18 ± 0.03 ^c^	0.18 ± 0.05 ^c^	0.41 ± 0.06 ^bc^	0.45 ± 0.13 ^bc^
Methional	0.053 ± 0.018 ^b^	0.00 ± 0.00 ^c^	0.00 ± 0.00 ^c^	0.041 ± 0.005 ^b^	0.00 ± 0.00 ^c^	0.083 ± 0.017 ^a^	0.00 ± 0.00 ^c^	0.050 ± 0.009 ^b^	0.041 ± 0.007 ^b^	0.00 ± 0.00 ^c^	0.00 ± 0.00 ^c^	0.00 ± 0.00 ^c^	0.00 ± 0.00 ^c^
Benzaldehyde	8.03 ± 0.61 ^a^	2.27 ± 0.13 ^c^	2.15 ± 0.28 ^c^	2.14 ± 0.22 ^c^	2.24 ± 0.73 ^c^	7.71 ± 0.20 ^ab^	2.67 ± 0.21 ^c^	5.91 ± 0.56 ^b^	6.29 ± 1.93 ^ab^	1.73 ± 0.23 ^c^	1.65 ± 0.40 ^c^	2.32 ± 0.24 ^c^	1.91 ± 0.28 ^c^
(*Z*)-6-Octen-2-one	1.83 ± 0.62 ^a^	1.54 ± 0.19 ^ab^	1.28 ± 0.17 ^abc^	0.133 ± 0.008 ^d^	0.93 ± 0.46 ^abcd^	0.63 ± 0.40 ^bcd^	0.61 ± 0.08 ^bcd^	0.14 ± 0.06 ^d^	1.04 ± 0.56 ^abcd^	1.05 ± 0.14 ^abcd^	0.73 ± 0.19 ^bcd^	1.24 ± 0.30 ^abc^	0.58 ± 0.12 ^cd^
(*E*,*E*)-2,4-Heptadienal	0.68 ± 0.17 ^a^	0.016 ± 0.005 ^b^	0.044 ± 0.035 ^b^	0.56 ± 0.11 ^a^	0.041 ± 0.006 ^b^	0.46 ± 0.11 ^ab^	0.029 ± 0.017 ^b^	0.67 ± 0.57 ^a^	0.77 ± 0.03 ^a^	0.019 ± 0.004 ^b^	0.039 ± 0.002 ^b^	0.037 ± 0.028 ^b^	0.032 ± 0.003 ^b^
Octanal	1.80 ± 0.19 ^a^	0.60 ± 0.03 ^cd^	0.49 ± 0.11 ^d^	1.27 ± 0.27 ^b^	0.46 ± 0.22 ^d^	1.21 ± 0.08 ^b^	0.53 ± 0.04 ^cd^	0.94 ± 0.07 ^bc^	1.91 ± 0.22 ^a^	0.39 ± 0.05 ^d^	0.47 ± 0.15 ^d^	0.68 ± 0.03 ^cd^	0.51 ± 0.15 ^cd^
2-Nonanone	1.27 ± 0.08 ^a^	1.05 ± 0.12 ^ab^	0.89 ± 0.17 ^abc^	0.12 ± 0.02 ^e^	0.54 ± 0.37 ^bcde^	0.53 ± 0.32 ^bcde^	0.255 ± 0.008 ^de^	0.13 ± 0.03 ^e^	0.83 ± 0.43 ^abcd^	0.40 ± 0.04 ^cde^	0.21 ± 0.07 ^e^	0.68 ± 0.18 ^bcde^	0.21 ± 0.05 ^e^
(*E*,*Z*)-2,6-Nonadienal	0.85 ± 0.09 ^a^	0.049 ± 0.006 ^d^	0.050 ± 0.012 ^d^	0.61 ± 0.09 ^b^	0.061 ± 0.037 ^d^	0.77 ± 0.10 ^ab^	0.054 ± 0.003 ^d^	0.42 ± 0.05 ^c^	0.91 ± 0.08 ^a^	0.055 ± 0.018 ^d^	0.053 ± 0.014 ^d^	0.061 ± 0.011 ^d^	0.065 ± 0.020 ^d^

Values within the same row with different letters (a–f) are significantly different *p* < 0.05 (Tukey test).

**Table 3:** S scores given by the panel members to the five conditions subjected to sensory analysis.

	pH 7 after 15-min Stirring	pH 7 + D30	pH 11 KOH after 15-min Stirring	pH 11 KOH + D30	pH 7 + D15 + pH 11 KOH + D15
S scores	88.0 ^a^	75.0 ^a^	51.0 ^b^	43.0 ^b^	43.0 ^b^

Values within the same row with different letters (a,b) are significantly different *p* < 0.05 (Wilcoxon).

## Data Availability

Data is contained within the article.
